# Effect of green tea consumption on blood lipids: a systematic review and meta-analysis of randomized controlled trials

**DOI:** 10.1186/s12937-020-00557-5

**Published:** 2020-05-20

**Authors:** Renfan Xu, Ke Yang, Sui Li, Meiyan Dai, Guangzhi Chen

**Affiliations:** 1 Department of Medical Ultrasound, Tongji Hospital, Tongji Medical College, Huazhong University of Science and Technology, Wuhan, 430030 People’s Republic of China; 2 Division of Cardiology, Department of Internal Medicine, Tongji Hospital, Tongji Medical College, Huazhong University of Science and Technology, Wuhan, 430030 People’s Republic of China

**Keywords:** Green tea, Catechin, Cholesterol, Triglycerides, Meta-analysis

## Abstract

**Background:**

Strong epidemiologic evidence indicates that green tea intake is protective against hyperlipidemia; however, randomized controlled studies have presented varying results. In the present study, we aimed to conduct a literature review and meta-analysis to assess the effect of green tea on blood lipids.

**Methods:**

PubMed, Embase, and the Cochrane Library were electronically explored from inception to September 2019 for all relevant studies. Random effect models were used to estimate blood lipid changes between green tea supplementation and control groups by evaluating the weighted mean differences (WMD) with 95% confidence intervals (CIs). The risk of bias for study was assessed using the Cochrane tool. Publication bias was evaluated using funnel plots and Egger’s tests.

**Results:**

Thirty-one trials with a total of 3321 subjects were included in the meta-analysis. In general, green tea intake significantly lowered the total cholesterol (TC); WMD: − 4.66 mg/dL; 95% CI: − 6.36, − 2.96 mg/dL; *P* < 0.0001) and low-density lipoprotein (LDL) cholesterol (WMD:− 4.55 mg/dL; 95% CI: − 6.31, − 2.80 mg/dL; *P* < 0.0001) levels compared with those in the control. Green tea consumption did not affect high-density lipoprotein (HDL) cholesterol; however, it reduced the triglycerides compared with that in the control (WMD: − 3.77 mg/dL; 95% CI: − 8.90, 1.37 mg/dL; *P* = 0.15). In addition, significant publication bias from funnel plots or Egger’s tests was not evident.

**Conclusions:**

Collectively, consumption of green tea lowers LDL cholesterol and TC, but not HDL cholesterol or triglycerides in both normal weight subjects and those who were overweight/obese; however, additional well-designed studies that include more diverse populations and longer duration are warranted.

## Introduction

Cardiovascular diseases (CVDs) are the leading cause of mortality and disability worldwide, accounting for approximately17.3 million deaths per year [1]. Hyperlipidemia, resulting from abnormalities due to lipid metabolism, causes atherosclerotic plaques and is considered a major risk factor for CVDs [2]. The previous study reports that subjects with hyperlipidemia have a three-fold risk of heart attack compared with those with normal lipid levels [3]. Moreover, CVDs risk was found to reduce by 3% when the serum cholesterol decreased by 1% [4]. Although several synthetic lipid-lowering medications (fibrates, statins, and bile acid sequestrants) are available in the market, their long-term usage might result in various adverse effects [5]. Agencies concerned with cardiovascular health have uniformly stressed the importance of lifestyle and diet as the primary means of lowering serum lipids and CVDs risk [6].

Green tea, which is derived from the plant *Camellia sinensis,* is a popular beverage worldwide, and can delay the onset or progression of numerous diseases such as cardiovascular disorders, metabolic diseases, and hypertension [7, 8]. Tea polyphenols, specifically catechins (flavonoids), are crucial in promoting health. The four major catechins (constituting 25–30%) reported in green tea are epicatechin (EC), epigallocatechin (EGC), epicatechingallate (ECG), and epigallocatechin gallate (EGCG) [9]. EGCG is most abundant (50–60% of total catechins), and has anti-inflammatory, antioxidant, anticarcinogenic, and antiobesity properties [10, 11]. Green tea also contains theaflavins, caffeine, phenolic acids, and flavonols such asquercetin, kaempferol, and myricetin [12].

Both in vitro and animal experiments have shown that green tea catechins can significantly reduce the levels of plasma triglycerides, total cholesterol (TC), and low-density lipoprotein (LDL) cholesterol [11, 12]. However, clinical trial results have not been conclusive regarding these effects of green tea. Some randomized controlled trials (RCTs) and meta-analyses have suggested that green tea may affect the lipid profiles in subjects with cardiovascular-related diseases such as hypercholesterolemia, hypertension, and glucose intolerance as well as in healthy individuals [13, 14], whereas other RCTs have not been able to confirm the positive metabolic effects of green tea [15, 16].

In this article, we report a systematic review and meta-analysis of RCTs to quantitatively assess the effect of green tea on total cholesterol, LDL, HDL, and triglyceride levels based on the Preferred Reporting Items for Systematic Reviews and Meta-Analyses (PRISMA) guidelines [17].

## Methods

### Search strategy and eligibility criteria

We explored PubMed, Embase, and the Cochrane Library from the index date of each database through September 2019 by using the following terms: “green tea,” “tea component(s),” “green tea extract,” “tea solid(s),” “catechins,” “EGCG,” “*Camellia sinensis,*” and “tea polyphenols,” which were paired with the following words: “blood lipid,” “blood cholesterol,” “high-density lipoprotein cholesterol,” “low-density lipoprotein cholesterol,” “triglyceride,” or “cardiovascular.” We further restricted the search to studies on humans and to English articles. Additional studies not captured by our database search were retrieved via a manual search of references from the originally identified reviews and research reports.

### Study selection

The prespecified inclusion criteria were as follows: 1) adult subjects who had ingested green tea for≥2 weeks; 2) use of an RCT design; 3) trial reported effects on TC,LDL cholesterol, HDL cholesterol, or triglycerides;4) green tea extract not being administered as part of a multicomponent supplement in either the experimental or control group; 5) the study used a concurrent control group; the only difference between the treatment and control groups was the use of green tea or green tea extract; and 6) each group in the trial enrolled > 10 participants. The exclusion criteria were as follows: 1) trials that enrolled children or pregnant women;2) trials in which green tea was included as part of a calorie-containing beverage, for example, milk or fruit juice. The data of multiple published reports from the same study population were included only once.

### Assessment of risk of bias in included studies

Two authors (CGZ and XRF) independently assessed the risk of bias of each study, using the Cochrane tool for assessing risk of bias [18]. Any disagreement was resolved by discussion between the third author (YK). The risk of bias tool addresses the following domains: Bias arising from the randomisation process; Bias due to deviations from intended interventions; Bias due to missing outcome data; Bias in measurement of the outcome; Bias in selection of the reported result and overall bias. For each study we categorized each domain as ‘low risk of bias’, ‘high risk of bias’ or ‘Some concerns’. The overall risk of bias generally corresponds to the worst risk of bias in any of the domains. However, if a study is judged to have “some concerns” about risk of bias for multiple domains, it might be judged as at high risk of bias overall.

### Data extraction

We extracted all data using a standardized data collection form. The following information was sought from each article: study characteristics (the first author, year of publication, study design, sample size, study duration, intervention type and dose), participant characteristics (age, sex, country, and baseline cholesterol status), and mean differences in the levels of TC, LDL, HDL, and triglycerides, representing the primary outcome measures. Two authors (CGZ and XRF) independently extracted the data, and any disagreements were resolved by discussion with a third author (YK). All values were converted to milligram per deciliters (mg/dL) in this trial. Although outcomes were reported several times at different stages of the trials, only the final outcome concentrations, at the end of the trial, were included in the meta-analysis.

### Statistical analysis

We performed this meta-analysis by using STATA statistical software (version 11; STATA Corp LP). Treatment effects were defined as the mean differences and 95% confidence intervals (CIs) calculated for changes in TC, LDL, HDL, and triglyceride levels between the intervention and control groups from baseline to the end of the intervention period. Pooled estimated effects were calculated by assigning each study a weight of the reciprocal of its variance. If the standard deviations (SDs) were not reported directly, then the variances were imputed from *P* values, 95% CIs, standard error (SE), or *t* values [19]. In addition, missing SD values for paired differences were imputed by assuming a correlation coefficient of 0.5 between the variances at baseline and end of trial according to the method by Follmann et al. [20]. Random-effects models (DerSimonian and Laird), which considered both within- and between-study variation, were performed for the studies used different doses, different populations, different durations and so on.

Statistical heterogeneity was estimated by using Cochran’s test (*P* < 0.10 was considered statistically significant) and heterogeneity was quantified with the I^2^ statistic. *I*^2^ > 50% indicated significant heterogeneity across studies [21]. Prespecified subgroup analyses were performed according to: catechin dosage (≥615 mg/day, high median vs. < 615 mg/day, low median); green tea intervention duration (≥12 weeks, long-term vs. < 12 weeks, short-term); intervention type (green tea beverage or green tea capsule); participant ethnicity (Asian or Western countries); study design (parallel or crossover); health status of participants (healthy subjects vs. obese subjects). Furthermore, meta-regression analysis was performed to examine the association between the net change in serum lipids and intervention dose, treatment duration, intervention type, caffeine content, different ethnicity and study design. Sensitivity analyses were performed to assess the stability of the results by removing one study each time to identify the impact of individual studies on the pooled effect size. Funnel plots and Egger’s regression test were used to assess the publication bias [22]. A *P* value of < 0.05 was considered statistically significant in this trial, unless otherwise specified.

## Results

### Results of the literature search

**Fig. 1:**
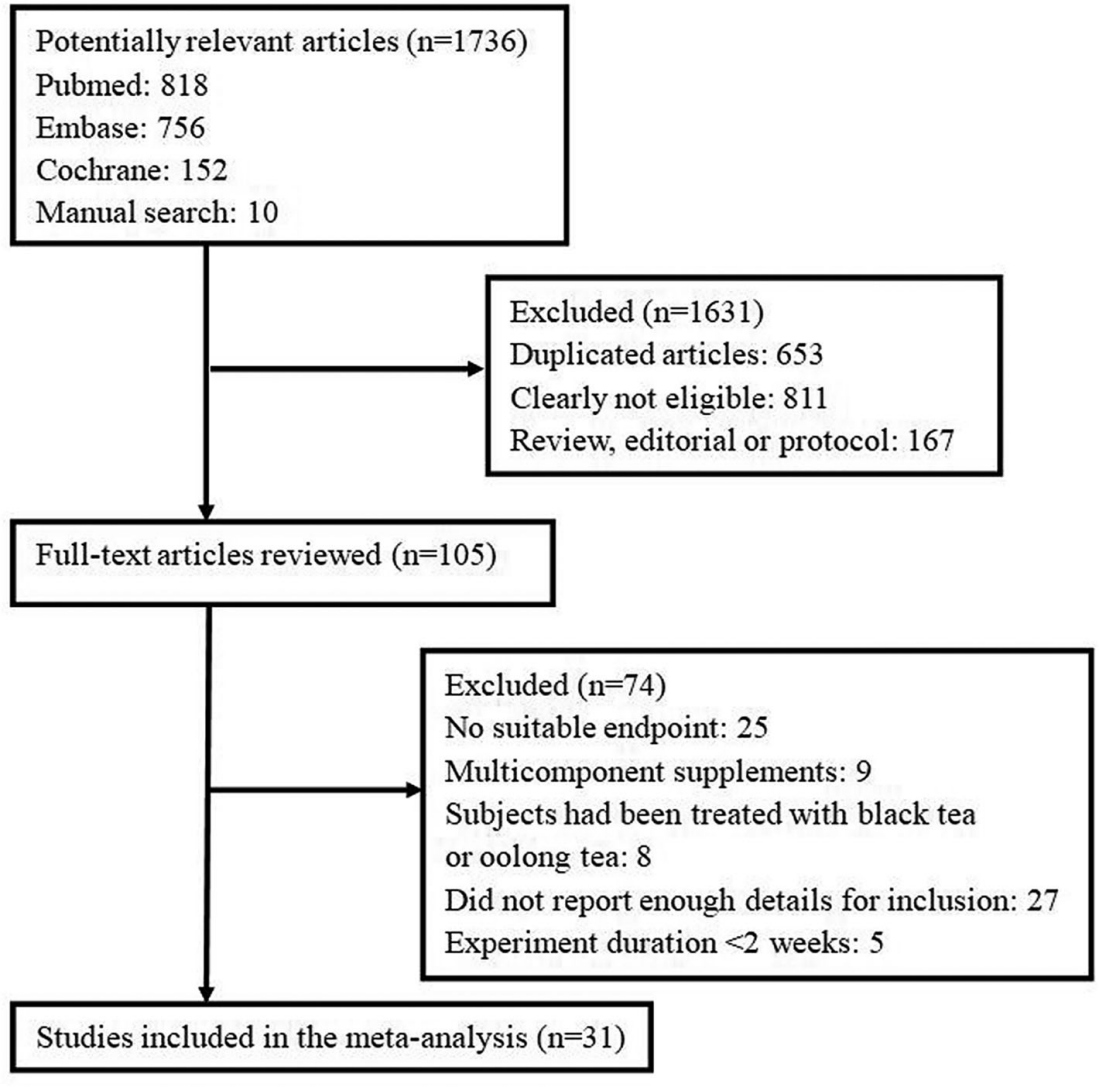
Flow diagram of the study selection procedure presenting the number of eligible articles included in the meta-analysis

The detailed process of the study selection is depicted in Fig. 1. In total, 1736 potentially relevant articles were initially identified from PubMed, EMBASE, and the Cochrane Library, collectively with manually searched articles. A total of 1631 articles were excluded, either because of duplication or because they were deemed irrelevant on the basis of the article title and abstract screening. We included 105 articles in the full-text review during which 74 articles were excluded for various reasons: 27 articles did not report enough details for inclusion, 25 articles did not report relevant outcomes, 8 articles were excluded because the subjects had been treated with black tea or oolong tea, 5 studies were < 2 weeks in duration, 9 studies used green tea a multicomponent supplement in the experimental group. Thus, 31 articles were eventually selected for inclusion in the meta-analysis.

### Study characteristics

Thirty-one eligible RCTs with a total of 3216 subjects were enrolled in the meta-analysis [23–53]. Trial characteristics are summarized in Table 1. In these trials, the study duration ranged from 3 weeks to 12 months, trial size varied from 20 to 936 subjects, green tea catechin intake in the intervention groups ranged from 80 to 2488.7 mg/d. We included 31 trials with 33 comparisons in this meta-analysis. Thirty-one comparisons reported on total cholesterol (*n* = 3024 subjects) [23–36, 38–40, 42–53], 29 comparisons reported on LDL cholesterol (*n* = 3005) [23–29, 32–40, 42–44, 46–53], 29 comparisons reported on HDL cholesterol (*n* = 3073) [23–30, 32–36, 38–44, 47–53] and 29 comparisons reported on outcomes for triglycerides (*n* = 3025 subjects) [23–29, 31–36, 38–41, 43–45, 47–53]. Sixteen comparisons were conducted in western countries [23–26, 29–31, 40, 42, 46–48, 50, 53] and 17 comparisons were conducted in Asian countries [27, 28, 32–39, 41, 43–45, 49, 51, 52]. Twelve comparisons were performed in healthy normal weight subjects [31, 36, 39, 43, 46–49, 52, 53], and 21 comparisons were conducted in over-weight to obese patients [23–30, 32–35, 37, 38, 40–42, 44, 45, 50, 51]. Most of the comparisons (30/33) used a parallel study design [23–25, 27–31, 33, 34, 36–53], whereas 3 comparisons adopted a crossover design [26, 32, 35]. Fourteen comparisons ruled out the confounding effect of caffeine on lipid concentrations [23, 25, 26, 28, 34, 35, 37–39, 42, 46, 48, 53], 12 comparisons used caffeinated green tea as supplements [27, 29–33, 40, 43–45, 49, 52] and 7 did not report the use of coffee [24, 36, 41, 47, 50, 51]. Nine comparisons selected green tea beverage [23, 32, 40, 43–45, 47, 49, 52], and 24 comparisons used green tea extract capsule [24–31, 33–39, 41, 42, 46–48, 50, 51, 53] (Table 1).

**Table 1:** Characteristics of 31 included randomized controlled trials

Reference	Study design	No.of subjects (M/F)	Age (y)	Country or Region	Population	Duration	Tea group	Control group
Basu 2011 [23]	P	25(5/20)	43.7 ± 3	USA	Obese,	8wk	decaffeinated GTE beverage (928 mg catechins)	Placebo (water)
Bogdanski 2012 [24]	P	56(28/28)	50.4 ± 8	Poland	Obese	3mo	GTE capsule(208 mg EGCG)	placebo (cellulose)
Brown 2009 [25]	P	88(88/0)	51.4 ± 6.4	UK	Overweight/obese	8wk	decaffeinated GTE capsule (800 mg EGCG)	Placebo (lactose)
Brown 2011 [26]	C	66(66/0)	49.5 ± 5.6	UK	Overweight/obese	6wk	decaffeinated GTE capsule (800 mg catechins)	Placebo (lactose)
Chan 2006 [27]	P	34(0/34)	34.8 ± 4.2	China	obese	3mo	GTE capsule (661.3 mg cathchins, 152.8 caffeine)	placebo
Chen 2016 [28]	P	77(0/77)	44.5 ± 11.5	Taiwan	Obese	12wk	decaffeinated GTE capsule (1344 catechins)	placebo (cellulose)
Diepvens 2006 [29]	P	46(0/46)	41.6 ± 10	Netherlands	Overweight	12wk	GTE capsule (1125 mg catechins, 225 mg caffeine)	placebo
Frank 2009 [30]	P	33(33/0)	40.5 ± 10	UK	Overweight	3wk	GTE capsule (672 mg catechins, 114 mg caffeine)	Placebo (matched with caffeine)
Freese 1999 [31]	P	20(0/20)	23–50	Finland	Healthy	4wk	GTE capsule (630 mg cathchins,150 caffeine)	Placebo (saccharose, microcrystalline, cocoa)
Fukino 2008 [32]	C	60(49/11)	53.6 ± 8.2	Japan	Diabetes, overweight	2mo	GTE beverage (456 mg catechins, 102 mg caffeine)	No intervention
Hsu 2008 [33]	P	78(0/78)	43.5 ± 12	Taiwan	Obese	3mo	GTE capsule (613.5 mg cathchins, 27.3 caffeine)	Placebo
Hsu 2011 [34]	P	68(24/44)	51.4 ± 9.2	Taiwan	Obese, Diabetes	16wk	decaffeinated GTE capsule (1344 catechins)	placebo (cellulose)
Huang 2018 [35]	C	73(0/73)	55 ± 9.5	Taiwan	Overweight/obese	6wk	decaffeinated GTE capsule (1344 catechins)	Placebo (microcrystalline cellulose)
Kafeshani 2017 [36]	P	32(32/0)	21 ± 2	Iran	Healthy	6wk	GTE capsule (240 mg catechins)	placebo (maltodextrin)
Lee 2016 [37]	P	77(66/11)	62 ± 12	Taiwan	Chronic Stable Angina, overweight	6wk	decaffeinated GTE capsule (600 mg polyphenol)	placebo
Liu 2014 [38]	P	77(32/45)	54.3 ± 7	Taiwan	Diabetes, obese	16wk	decaffeinated GTE capsule (1344 catechins)	placebo (cellulose)
Lu 2016 [39]	P	64(0/64)	29 ± 10	Taiwan	Acne	4wk	decaffeinated GTE capsule (1344 catechins)	placebo (cellulose)
Maki 2009 [40]	P	128(67/61)	48 ± 9	USA	Obese	12w	GTE beverage (625 mg catechins, 39 mg caffeine)	Placebo (matched with caffeine)
Maron 2003 [41]	P	240(100/140)	54.7 ± 11	China	Hypercholesterolemia, overweight	12wk	GTE capsule (150 mg cathchins)	Placebo (inertingredients)
Mielgo-Ayuso 2014 [42]	P	83(0/83)	18–49	Spain	Obese	12wk	300 mg EGCG	Placebo (lactose)
Miyazaki 2013 [43]	P	52(20/32)	69.1 ± 5.9	Japan	Not obese	14wk	GTE beverage (630.9 mg catechins, 77 mg caffeine)	GTE beverage (88.7 mg catechins and 82.4 mg caffeine)
Nagao 2007 [44]	P	240(140/100)	41.7 ± 9.9	Japan	Overweight	12wk	GTE beverage (582.8 mg catechins, 72.3 caffeine)	GTE beverage (96 mg catechins, matched with caffeine)
Nagao 2009 [45]	P	43(18/25)	63.9 ± 2	Japan	Diabetes, overweight	12wk	GTE beverage (582.8 mg catechins, 72.3 caffeine)	GTE beverage (96 mg catechins, matched with caffeine)
Nantz 2009 [46]	P	111 (46/65)	29 ± 10.9	USA	Healthy	3mo	decaffeinated GTE capsule (320 catechins)	Placebo (maltodextrin)
Princen-a 1998 [47]	P	30	33.5 ± 13	Netherlands	Healthy	4wk	GTE beverage (851.7 mg catechins)	Placebo (mineral water)
Princen-b 1998 [47]	P	28	34 ± 12	Netherlands	Healthy	4wk	GTE capsule (2488.7 mg catechins)	Placebo (mineral water)
Samavat 2016 [48]	P	936(0/936)	60 ± 5	USA	Healthy	12mo	decaffeinated GTE capsule (1315 mg catechins)	Placebo (maltodextrin and cellulose)
Sone 2011 [49]	P	51(18/33)	45.7 ± 13.6	Japan	Healthy	9wk	GTE beverage (400 mg catechins, 105 mg caffeine)	GTE beverage (100 mg catechins; 80 mg caffeine)
Suliburska 2012 [50]	P	46(23/23)	50.4 ± 8.3	Poland	Obese	3mo	GTE capsule (208 mg EGCG)	placebo (cellulose)
Tadayon 2018 [51]	P	79(0/79)	53.3 ± 4	Iran	Overweight/ obese	4wk	800 mg GTE capsule (80-94polyphenol)	placebo
Venkatakrishnan 2018 [52]	P	40	NA	Taiwan	Healthy	12wk	GTE beverage (780.6 mg of catechin,106.7 caffeine)	placebo
Wu-a 2012 [53]	P	69(0/69)	58.7 ± 6.4	USA	Healthy	8wk	decaffeinated GTE capsule (536 mg catechins)	placebo
Wu-b 2012 [53]	P	66(0/66)	59.9 ± 7.9	USA	Healthy	8wk	decaffeinated GTE capsule (1072 mg catechins)	placebo

*GTE* green tea extract, *P* parallel trial, *C* crossover trial, *wk.* week, *mo* month, *NA* not available

### Risk of bias

**Fig. 2:**
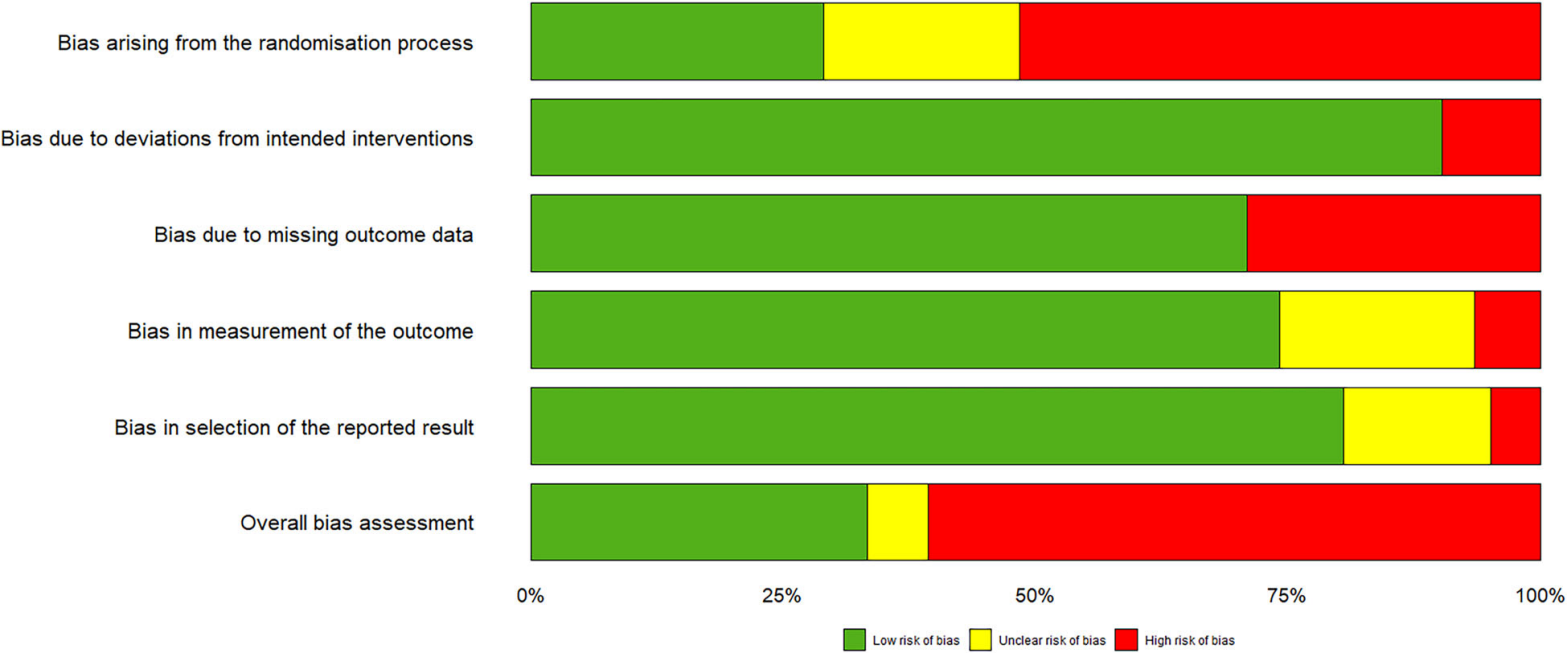
Risk of bias summary: review authors’ judgements about each risk of bias item for included studies

Overall nine of the 31 studies were at low overall risk of bias with no items at unclear or high risk of bias [25, 26, 28, 33, 34, 39, 42, 48, 51]; two were at unclear risk of bias with no items at high risk of bias [35, 52]; and 20 were at high risk of bias [23, 24, 27, 29–32, 36–38, 40, 41, 43–47, 49, 50, 53] (Fig. 2). The risk of bias judgments and the details for each trial are in supplementary Table 1.

### Main outcomes

**Fig. 3:**
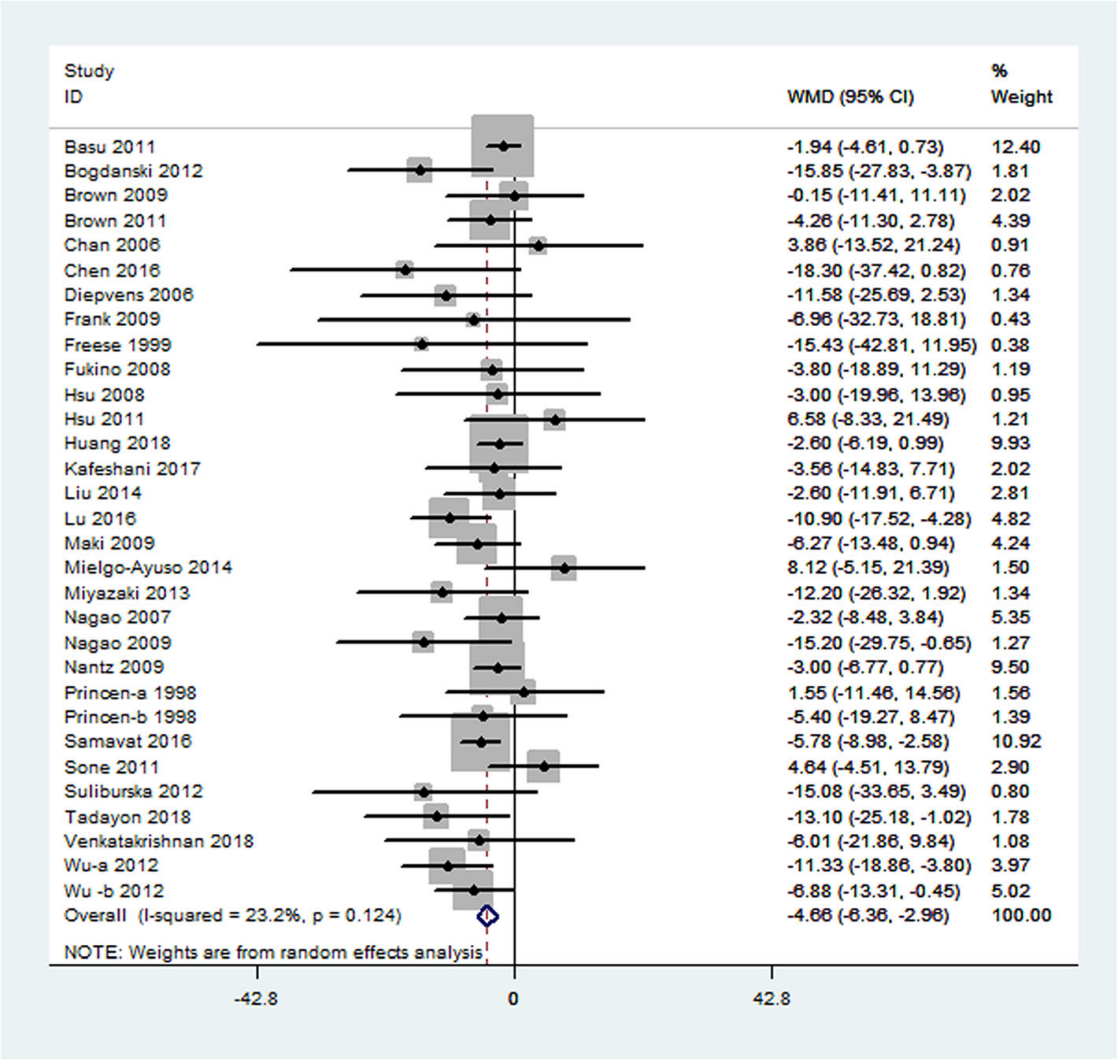
Meta-analysis of the effects of green tea on total cholesterol concentrations. Results from individual trials were pooled with the use of random-effect models and are expressed as weighted mean differences with 95% CIs

Primary outcome measures included changes in TC, LDL cholesterol, HDL cholesterol, and triglycerides. Thirty-one studies representing 3024 participants reported results for serum TC concentrations. Collectively, a significant difference was observed in the serum TC level in the green tea supplementation and the control groups (weighted mean difference: − 4.66 mg/dL; 95% CI: − 6.36, − 2.96 mg/dL; *P* < 0.0001). This difference represents a 2.3% decrease in the TC concentration while consuming green tea. Heterogeneity was not significant for this outcome (I^2^ = 23.2%, *P* = 0.124; Fig. 3).

**Fig. 4:**
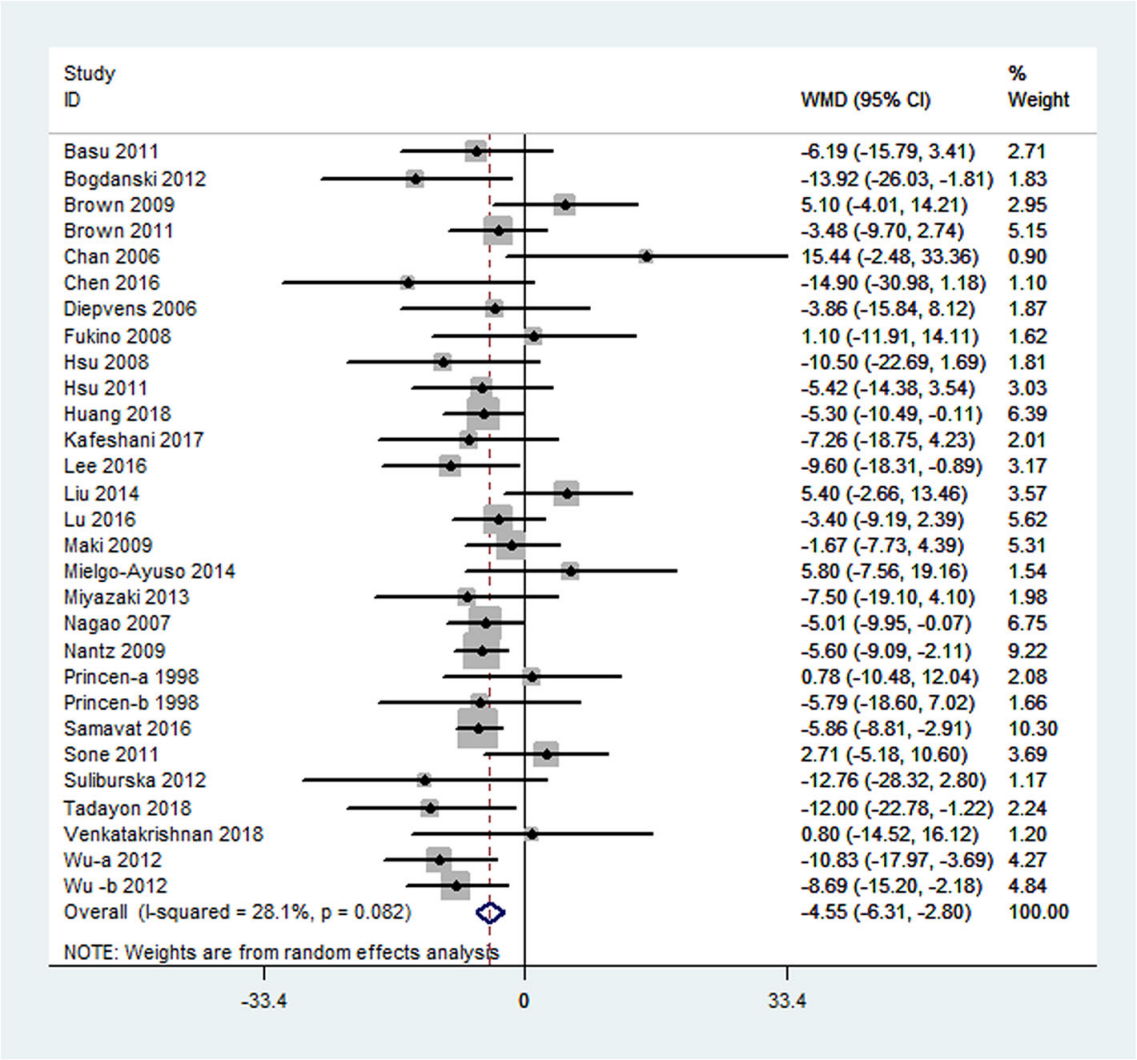
Meta-analysis of the effects of green tea on LDL cholesterol concentrations. Results from individual trials were pooled with the use of random-effect models and are expressed as weighted mean differences with 95% CIs. LDL cholesterol: low-density lipoprotein cholesterol

Results for LDL cholesterol were reported in 29 studies representing 3005 participants. Green tea supplementation significantly reduced the LDL cholesterol by − 4.55 mg/dL (95% CI: − 6.31, − 2.80 mg/dL; *P* < 0.0001) compared with the placebo effects. The degree of heterogeneity was significant (I^2^ = 28.1%; *P* = 0.082) (Fig. 4).

**Fig. 5:**
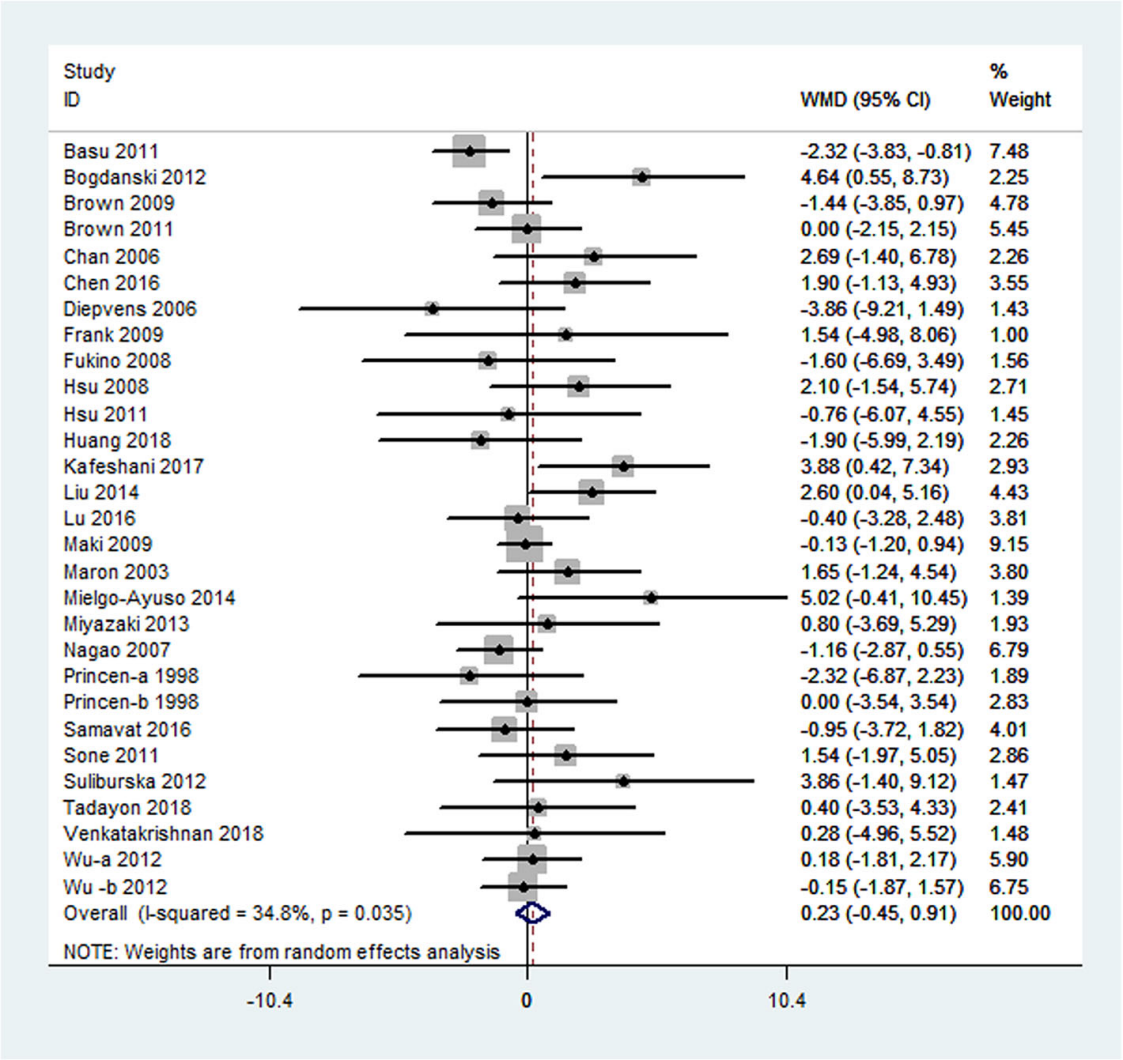
Meta-analysis of the effects of green tea on HDL cholesterol concentrations. Results from individual trials were pooled with the use of random-effect models and are expressed as weighted mean differences with 95% CIs. HDL cholesterol: high-density lipoprotein cholesterol

The mean change in HDL cholesterol concentrations was reported in 29 studies, which represented 3073 participants. In general, no significant difference was observed in serum HDL between the green tea supplementation and placebo groups (weighted mean difference: 0.23 mg/dL; 95% CI: − 0.45, 0.91 mg/dL; *P* = 0.50). The overall result for the heterogeneity test was significant (I^2^ = 34.8%; *P* = 0.035) (Fig. 5).

**Fig. 6:**
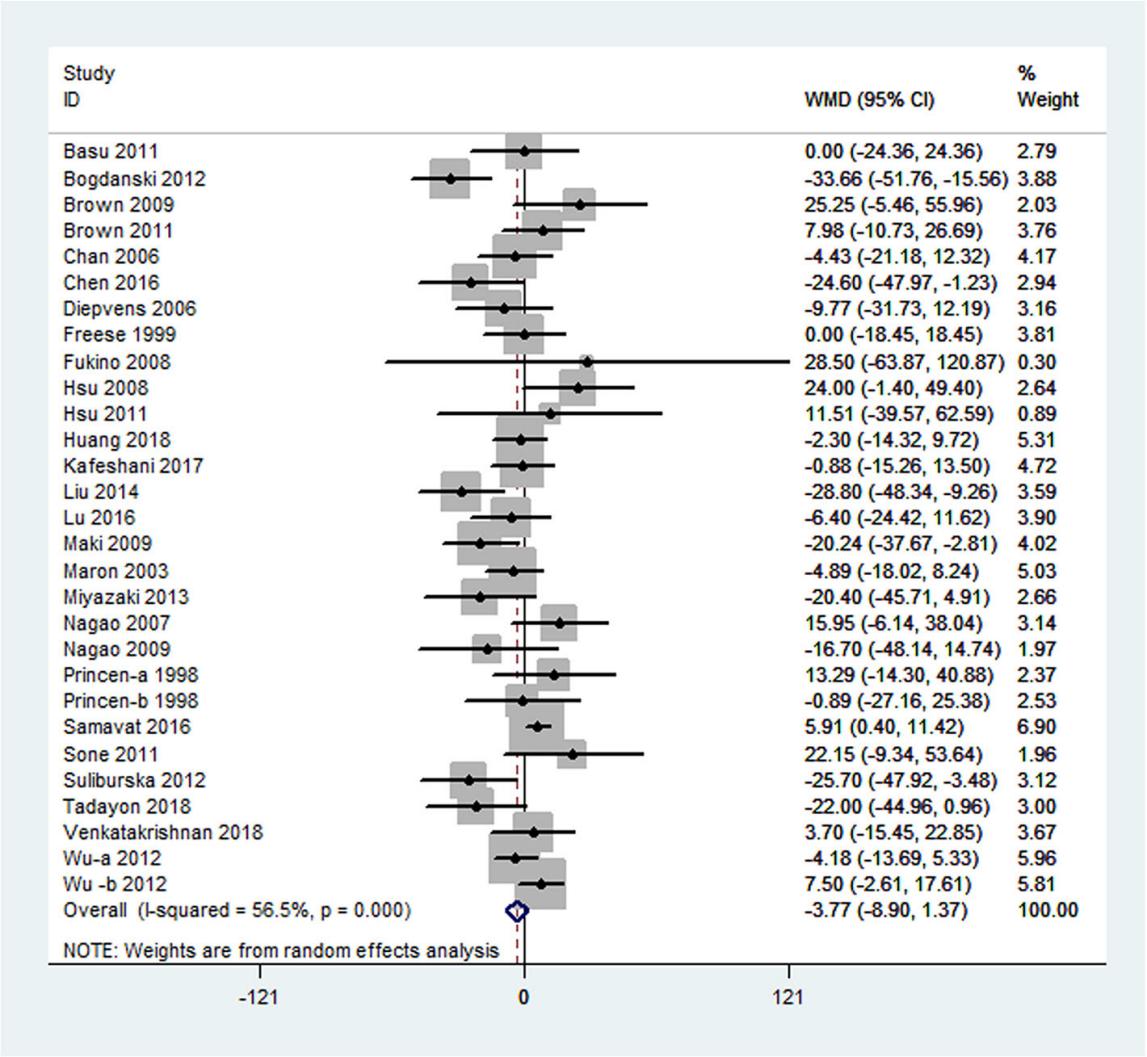
Meta-analysis of the effects of green tea on triglyceride concentrations. Results from individual trials were pooled with the use of random-effect models and are expressed as weighted mean differences with 95% CIs

Serum triglyceride concentrations were calculated in 29 comparisons that included 3025 subjects. Although differences in triglyceride levels did not attain statistical significance, we observed a trend in favor of green tea − 3.77 mg/dL (95% CI: − 8.90, 1.37 mg/dL; *P* = 0.15). Large heterogeneity (I^2^ = 56.5%; *P* = 0.0001) was observed in this outcome (Fig. 6).

### Subgroup analysis and meta-regression

In the subgroup analysis, the beneficial effect of green tea intake on total cholesterol was consistently observed in all the analyses except for the study design subgroups. Green tea consumption significantly lowered TC in subgroups with parallel design, but no effect was found in the subgroup with crossover design. In addition, the beneficial effect of green tea intake on LDL cholesterol was also consistently observed in most subgroup analyses except for the decaffeination subgroup. A significant reduction in LDL cholesterol was observed in the decaffeination subgroup; however, no effect was observed in the caffeine subgroup. Meta-analysis indicated that green tea has no effect on serum HDL cholesterol, which was consistent in all the subgroup analyses. Subgroup analysis suggested that triglycerides were reduced to a greater degree in the studies with longer duration subgroup, with mean changes of − 9.03 mg/dL (95% CI: − 17.92, − 0.15 mg/dL; *P* = 0.04); however, no effect of green tea on triglycerides was observed in other subgroup analysis (Table 2).

**Table 2:** Subgroup analyses of mean change in total cholesterol, LDL cholesterol, HDL cholesterol and triglyceride

Subgroup	Change in total cholesterol			Change in LDL cholesterol			Change in HDL cholesterol			Change in Triglyceride		
	Trials (n)	Net change (95% CI) (mg/dl)	I^2^	Trials (n)	Net change (95% CI) (mg/dl)	I^2^	Trials (n)	Net change (95% CI) (mg/dl)	I^2^	Trials (n)	Net change (95%CI) (mg/dl)	I^2^
Type of intervention												
Green tea beverage	9	−2.80(−5.43, −0.16)	10%	8	−2.61(− 5.49, 0.27)	0%	8	− 0.84(− 1.74, 0.06)	17%	9	− 0.77(− 12.15, 10.61)	42%
Green tea capsule	22	−5.41(−7.46, −3.37)	21%	21	− 5.28(− 7.44, − 3.12)	36%	21	0.73(− 0.07, 1.54)	25%	20	− 4.62(− 10.52, 1.27)	62%
Duration												
≥ 12 weeks	15	−5.59(−8.66, − 2.51)	23%	14	− 4.04(− 7.13, − 0.94)	39%	15	0.92(− 0.12, 1.96)	39%	15	−9.03(− 17.92, − 0.15)	72%
< 12 weeks	16	− 4.05(− 6.05, − 2.06)	21%	15	− 4.91(− 7.06, − 2.75)	20%	14	− 0.42(− 1.26, 0.41)	20%	14	0.77(− 3.83, 5.36)	3%
Country												
Western	16	− 4.79(− 6.89, − 2.70)	24%	14	−5.07(− 7.25, − 2.89)	23%	14	−0.25(− 1.18, 0.68)	43%	13	− 3.11(− 10.72, 4.49)	66%
Asian	15	−4.50(− 7.58, − 1.43)	27%	15	−3.98(− 6.87, − 1.09)	34%	15	0.79(− 0.13, 1.71)	12%	16	−4.52(− 11.50, 2.46)	42%
Catechins dose												
≥ 642 mg/dl	16	−4.44(− 6.52, − 2.36)	22%	15	−3.53(− 6.14, − 0.92)	36%	16	−0.34(− 1.23, 0.55)	26%	15	−1.89(− 7.70, 3.93)	41%
< 642 mg/dl	15	−5.14(− 8.17, − 2.11)	29%	14	−5.52(− 7.93, − 3.10)	20%	13	0.92(− 0.11, 1.95)	39%	14	−5.75(− 14.99, 3.49)	66%
Caffeine												
With caffeine	12	−4.28(− 7.61, − 0.96)	0%	9	− 2.33(− 5.69, 1.04)	16%	10	−0.11(− 0.90, 0.68)	0%	11	− 1.20(− 10.48, 8.08)	42%
Without caffeine	13	−4.27(− 6.44, − 2.10)	42%	14	−4.81(− 7.04, − 2.57)	38%	12	−0.17(−1.16, 0.82)	43%	11	−1.18(− 8.07, 5.71)	57%
unclear	6	−8.17(−13.84, − 2.49)	12%	6	−8.15(−13.08, −3.22)	0%	7	1.72(0.00, 3.44)	29%	7	−11.22(−22.82, 0.38)	60%
Study design												
Parallel	28	−5.04(− 7.03, −3.04)	29%	26	−4.64(− 6.63, −2.64)	34%	26	0.37(−0.38, 1.11)	40%	26	−4.47(− 10.10, 1.15)	60%
Crossover	3	−2.98(− 6.11, 0.15)	0%	3	−4.07(− 7.88, −0.26)	0%	3	−0.56(− 2.34, 1.22)	0%	3	1.03(−9.02, 11.08)	0%
Health status												
Overweight or obese	19	−3.86(−6.11, −1.61)	19%	18	−3.97(−6.75, − 1.18)	39%	19	0.34(−0.62, 1.30)	49%	18	−7.63(−15.53, 0.28)	58%
Normal weight	12	−5.59(−8.07, −3.10)	22%	11	−5.46(−7.23, −3.69)	0%	10	0.17(−0.73, 1.08)	0%	11	2.56(−1.57, 6.69)	7%
Study Bias												
Low risk	9	−5.13(−9.15, −1.11)	41%	9	−4.49 (−7.64, −1.34)	33%	9	0.12(−0.90, 1.15)	0%	8	1.23(−9.66, 12.13)	59%
Some concerns	2	−2.77(−6.27, 0.74)	0%	2	−4.67(−9.59, 0.25)	0%	2	−1.07(−4.30, 2.15)	0%	2	−0.60(−10.78, 9.57)	0%
High risk	20	−4.53(−6.66, −2.41)	18%	18	−4.56 (−7.07, −2.05)	36%	18	0.39(−0.55, 1.33)	50%	19	−6.33(−12.83, 0.16)	54%

**Fig. 7:**
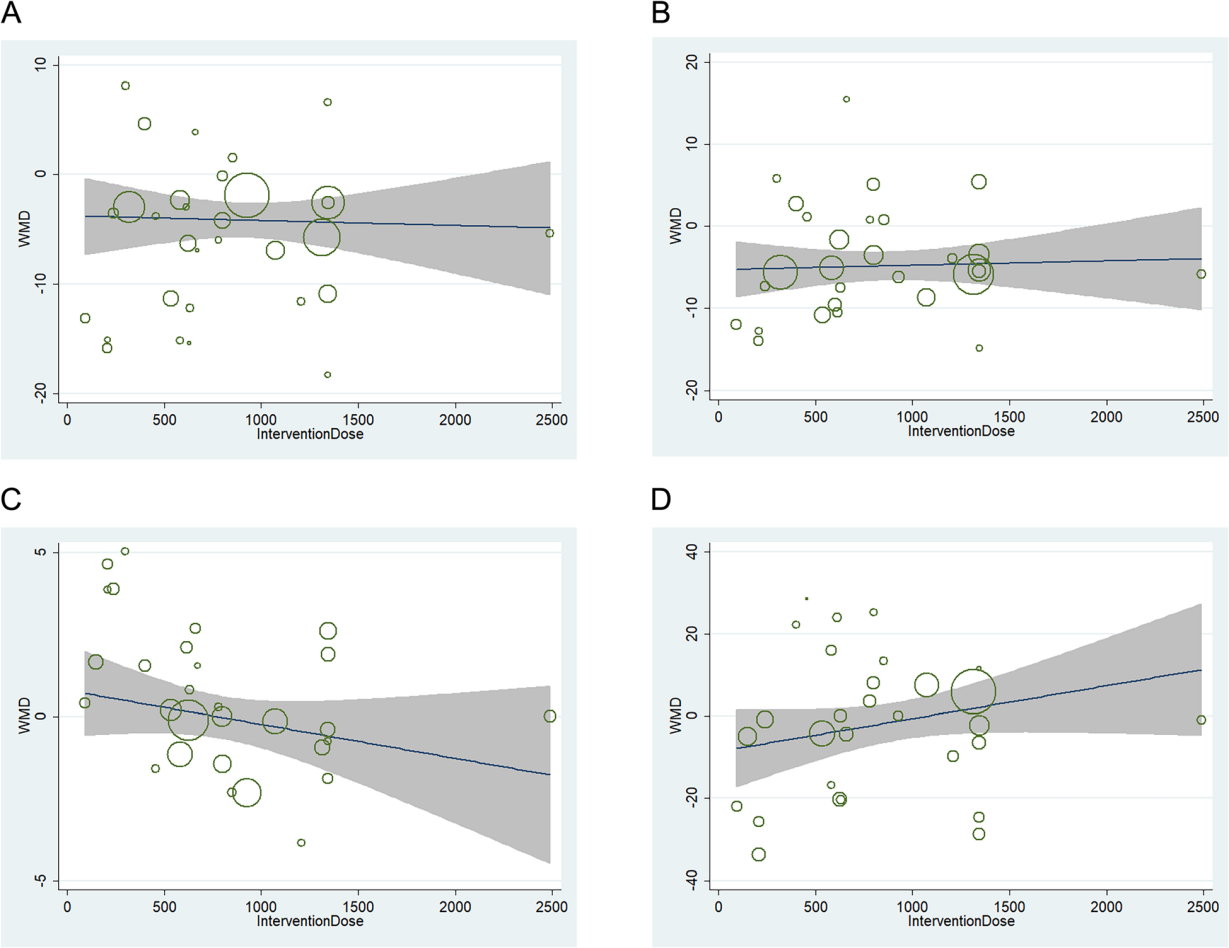
**a** Relation between the WMD of total cholesterol and intervention dose in 31 independent randomized controlled comparisons. **b** Relation between the WMD of LDL cholesterol and intervention dose in 29 independent randomized controlled comparisons. **c** Relation between the WMD of HDL cholesterol and intervention dose in 29 independent randomized controlled comparisons. **d** Relation between the WMD of triglyceride and intervention dose in 29 independent randomized controlled comparisons. Each circle represents a study, telescoped by its weight in the analysis. Meta-regression found no linear relations between WMD in TC (*p* = 0.94), LDL cholesterol (*p* = 0.69), HDL cholesterol (*p* = 0.11) or triglycerides(*p* = 0.49) and intervention dose

Meta-regression found no linear relations between net change in TC, LDL cholesterol, HDL cholesterol or triglycerides and intervention dose (Fig. 7). Furthermore, meta-regression found no linear relations between net change in serum lipid and treatment duration, caffeine content, different ethnicity, intervention type and study design.

### Publication bias

The funnel plots of the studies were symmetrical for TC, LDL cholesterol, HDL cholesterol, and triglyceride (supplementary Figure 1). Furthermore, Egger’s test suggested that no strong evidence was seen for a publication bias for TC (*P* = 0.63), LDL cholesterol (*P* = 0.54), HDL cholesterol (*P* = 0.43), or triglycerides (*P* = 0.36).

A sensitivity analysis was performed to confirm the robustness of our findings, in which one study at a time was excluded and the rest were analyzed; herein, the pooled reductions in TC ranged from − 4.77 mg/dL (95% CI: − 6.40, − 3.14 mg/dL) to − 4.26 mg/dL (95% CI: − 5.90, − 2.63 mg/dL); the pooled reductions in LDL cholesterol ranged from − 4.85 mg/dL (95% CI: − 6.57, − 3.13 mg/dL) to − 4.29 mg/dL (95% CI: − 6.04, − 2.54 mg/dL); the pooled reductions in HDL cholesterol ranged from 0.09 mg/dL (95% CI: − 0.56, 0.75 mg/dL) to 0.37 mg/dL (95% CI: − 0.26, 0.99 mg/dL); and the pooled reductions in triglyceride ranged from − 4.50 mg/dL (95% CI: − 9.61, 0.61 mg/dL) to − 2.49 mg/dL (95% CI: − 7.27, 2.30 mg/dL); removing a single trial did not hamper the study significance.

## Discussion

The present meta-analysis evaluated the association between green tea consumption and reduction in serum lipid concentrations based on published results from 31 studies comprising 3216 subjects. The results suggest that green tea supplementation significantly lowered both serum TC and LDL cholesterol concentrations. In addition, we demonstrated a trend toward decrease in triglyceride concentrations, although it did not attain significance, presumably because of the limited participants or duration for which the triglyceride concentrations were reported; however, green tea did not significantly affect the levels of HDL cholesterol. These findings are generally in accordance with the results from previous meta-analyses, which also identified a significant correlation between green tea supplementation and improvements in TC and LDL cholesterol concentration [13].

Recent mechanistic studies have examined the effects of green tea intake on lipid control and provide further evidence for the biological plausibility of these findings. In accordance with our results, several animal studies have reported that green tea supplementation significantly improved hyperlipidemia status in high-fat diet induced rats, including lowering TC, LDL cholesterol, and triglycerides [54]. Moreover, recent animal studies have indicated that green tea catechins could significantly inhibit atherosclerotic plaque formation, lower liver fat accumulation, and increase HDL cholesterol in hyperlipidemic rats induced by high-fat andhigh-cholesterol diet [55]. The mechanism underlying the beneficial effect of green tea on lipid control may be attributed to the high concentration of green tea catechins, which involve the following aspects:

(1) EGCG could attenuate the endothelial dysfunction induced by oxidized-LDL via the Jagged-1/Notch signaling pathway in human umbilical vein endothelial cells, which provides a beneficial effect by inhibiting the atherosclerotic plaque formation [56]. (2) Tea catechins are powerful antioxidants that prevent LDL oxidation by incorporating themselves into LDL particles in nonconjugated forms in vitro [57]*.* (3) These are responsible for LDL receptor binding activity upregulation in HepG2 cells in a dose-dependent manner by regulating the SREBP-1 (sterol regulatory binding protein-1) pathway [58]. (4) Green tea might also inhibit intestinal lipid absorption by interfering with micelle formation [59].

Observational studies have also indicated that green tea intake is inversely related to a risk of CVD. A large, 11-year population-based study involving > 40,000 middle-aged individuals from Japan revealed that, compared with non-tea drinkers, those with habitual green tea intake (over two cups daily, approximately 7 oz/day for 10 years) reduced their risk of death from CVD by 22–33% [7]. Randomized controlled trials have been performed to determine the effect of green tea on cholesterol concentration; however, the results are conflicting. Some studies have revealed that green tea intake significantly reduced the TC and LDL cholesterol [24, 53]. In contrast, several other studies reported no positive correlations between green tea intake and reduction in TC and LDL cholesterol [23, 52]. In addition, most studies suggested that no effect was seen for HDL cholesterol or triglycerides [35, 39], whereas only a few studies suggested a beneficial effect on HDL cholesterol or triglycerides levels [24].

The weighted mean reductions in TC and LDL cholesterol appearing due to green tea supplementation as observed in the present study (TC: 4.66 mg/dL; LDL cholesterol: 4.55 mg/dL), corresponding to reductions of 2–5%, might be important for primary prevention of cardiovascular health. Studies have reported that a 1% reduction in TC or LDL cholesterol was clinically associated with a 2–3% or 1% decreased risk of CVD, respectively [60]. Importantly, green tea intake did not negatively affect the serum HDL cholesterol levels. Thus, green tea supplementation mainly reduces the serum TC and LDL cholesterol concentrations but has limited effect on HDL cholesterol.

In this meta-analysis, subgroup analyses suggest that the beneficial effect of green tea was consistent in all the subgroup analyses except for the crossover design subgroups; however, only three trials were included in the crossover design, which were insufficient to make a significant conclusion. In addition, the beneficial effect of green tea intake on LDL cholesterol was also consistently observed in most subgroup analyses except for the caffeine subgroup. A significant reduction in LDL cholesterol was observed in the decaffeination subgroup instead of the caffeine subgroup. As caffeine is naturally present in green tea, whether caffeine intake affects lipid reduction is another potential issue, which continues to have conflicting opinions among previous studies [61, 62]. The meta-analysis indicated that green tea has no beneficial effect on serum HDL cholesterol, a finding that was consistent in all subgroup analyses. Green tea consumption has a beneficial effect on triglyceride levels in subjects with a longer duration of consumption (≥12 weeks); however, the benefit was not significant in other subgroup analyses. Because the number of trials available for subgrouping was limited, such analyses should be interpreted with caution. In addition, meta-regression found no significant relations between net change in serum lipid and intervention dose, treatment duration, caffeine content, different ethnicity, intervention type and study design. Larger and longer duration trials with optimally designed treatments and controls are required in the future research.

Our study has certain strengths. First, we only selected RCTs in this meta-analysis, which ensured relatively high-quality data and provided reliable inference about causality. Second, the relatively large number of pooled participants provided us higher statistical power to detect a small treatment effect. Third, results were unlikely to be influenced by publication bias. The results of Egger’s regression tests suggested no significant asymmetry of the funnel plot for the overall effect estimation of mean differences in TC, LDL cholesterol, HDL cholesterol, and triglyceride levels.

Our analyses did have a few limitations. First, the studies had relatively short duration of follow-up, ranging from 3 weeks to 12 months, with a median of 11 weeks, so presumed health benefits cannot be extrapolated beyond the duration of these studies; however, long-term effects are clinically important for lipid profiles and other CVD risk factors. Second, although considerable lowering of TC and LDL cholesterol by green tea intake was observed in our study, we could not determine the optimal dosage of green tea supplementation that would have the greatest impact on improving lipid metabolism, as the catechin dosage varied from 80 to 2488.7 mg/day (median: 630.9 mg/day). Third, our meta-analysis did not recognize a safety margin in this study, however, in some studies; concern has been raised as to the safety of high-dose green tea catechin supplementation. Mild side effects were reported in some clinical studies, including gastric upset, mild skin rashes, and abdominal bloating [26, 33]. In addition, green tea was known to be the major dietary source of oxalate in some patients with kidney oxalate stones [63]. Fourth, we identified large variations in study designs, catechin dosage, ethnic groups, green tea type, baseline health status, and trial quality. Although we did not identify these variations as statistically significant sources of heterogeneity, such heterogeneity may limit the validity of the overall pooled results. In addition, the articles included were all published in English; limited resources prevented us from including articles published in other languages.

## Conclusions

In conclusion, the results of this study indicate that green tea supplementation has a beneficial effect on TC and LDL cholesterol levels in both normal weight subjects and overweight/obese subjects; however, the protective role of green tea against high triglyceride levels was not supported in this study. Additional large prospective cohort studies are needed to provide a more definitive conclusion on the association between routine consumption of green tea and lipid metabolism.

## Supplementary information

**Additional file 1: Table S1.** Risk of bias for each included studies.

**Additional file 2: Figure S1.** A. Funnel plot of green tea supplementation and total cholesterol. B. Funnel plot of green tea supplementation and LDL cholesterol. C. Funnel plot of green tea supplementation and HDL cholesterol. *D. funnel* plot of green tea supplementation and triglyceride.

## Data Availability

All data generated or analyzed during this study are included in this published article.

## Abbreviations

BP: Blood pressure
CIs: Confidence intervals
CVDs: Cardiovascular diseases
TC: Total cholesterol
LDL: Low-density lipoprotein
HDL: High-density lipoprotein
EGCG: Epigallocatechin gallate
EC: Epicatechin
EGC: Epigallocatechin
ECG: Epicatechingallate
GTE: Green tea extracts
MDs: Mean differences
PRISMA: Preferred Reporting Items for Systematic Reviews and Meta-Analyses
RCTs: Randomized placebo-controlled trials
SD: Standard Deviation
SE: Standard error
WMD: Weighted mean differences
