# Supplementary material for: Effect of green tea consumption on blood lipids: a systematic review and meta-analysis of randomized controlled trials
Source: Nutr J. 2020 May 20;19:48. doi: 10.1186/s12937-020-00557-5 (PMC7240975; doi:10.1186/s12937-020-00557-5)
Supplement: Supplementary file 1 — Additional file 1: Table S1. Risk of bias for each included studies. [file 12937_2020_557_MOESM1_ESM.docx]

**Supplementary table 1:** Risk of bias for each included studies.

| References | Bias arising from the randomisation process | Bias due to deviations from intended interventions | Bias due to missing outcome data | Bias in measurement of the outcome | Bias in selection of the reported result | Total bias |
| --- | --- | --- | --- | --- | --- | --- |
| Basu 2011 | ? | - | + | - | + | - |
| Bogdanski 2012 | - | + | + | + | + | - |
| Brown 2009 | + | + | + | + | + | + |
| Brown 2011 | + | + | + | + | + | + |
| Chan 2006 | ? | + | - | ? | - | - |
| Chen 2015 | + | + | + | + | + | + |
| Diepvens 2006 | - | + | - | + | + | - |
| Frank 2009 | - | + | - | + | ? | - |
| Freese 1999 | - | + | - | + | - | - |
| Fukino 2008 | - | - | + | ? | + | - |
| Hsu 2008 | + | + | + | + | + | + |
| Hsu 2011 | + | + | + | ? | + | + |
| Huang 2018 | ? | + | + | + | + | ? |
| Kafeshani 2017 | - | + | + | + | + | - |
| Lee 2016 | - | + | - | - | + | - |
| Liu 2014 | - | + | + | + | + | - |
| Lu 2016 | + | + | + | + | + | + |
| Maki 2009 | - | + | - | + | + | - |
| Maron 2003 | - | + | + | + | ? | - |
| Mielgo-Ayuso 2014 | + | + | + | + | + | + |
| Miyazaki 2013 | - | + | + | + | + | - |
| Nagao 2007 | - | + | + | + | + | - |
| Nagao 2009 | - | + | - | ? | ? | - |
| Nantz 2009 | ? | + | - | ? | ? | - |
| Princen 1998 | - | - | + | + | + | - |
| Samavat 2016 | + | + | + | + | + | + |
| Sone 2011 | - | + | + | + | + | - |
| Suliburska 2012 | ? | + | - | + | ? | - |
| Tadayon 2017 | + | + | + | + | + | + |
| Venkatakrishnan 2018 | ? | + | + | + | + | ? |
| Wu 2012 | - | + | + | + | + | - |

+：Low risk; ?: Some concern; - : High risk.
