# Supplementary figures and images for: Effect of green tea consumption on blood lipids: a systematic review and meta-analysis of randomized controlled trials
Source: Nutr J. 2020 May 20;19:48. doi: 10.1186/s12937-020-00557-5 (PMC7240975; doi:10.1186/s12937-020-00557-5)

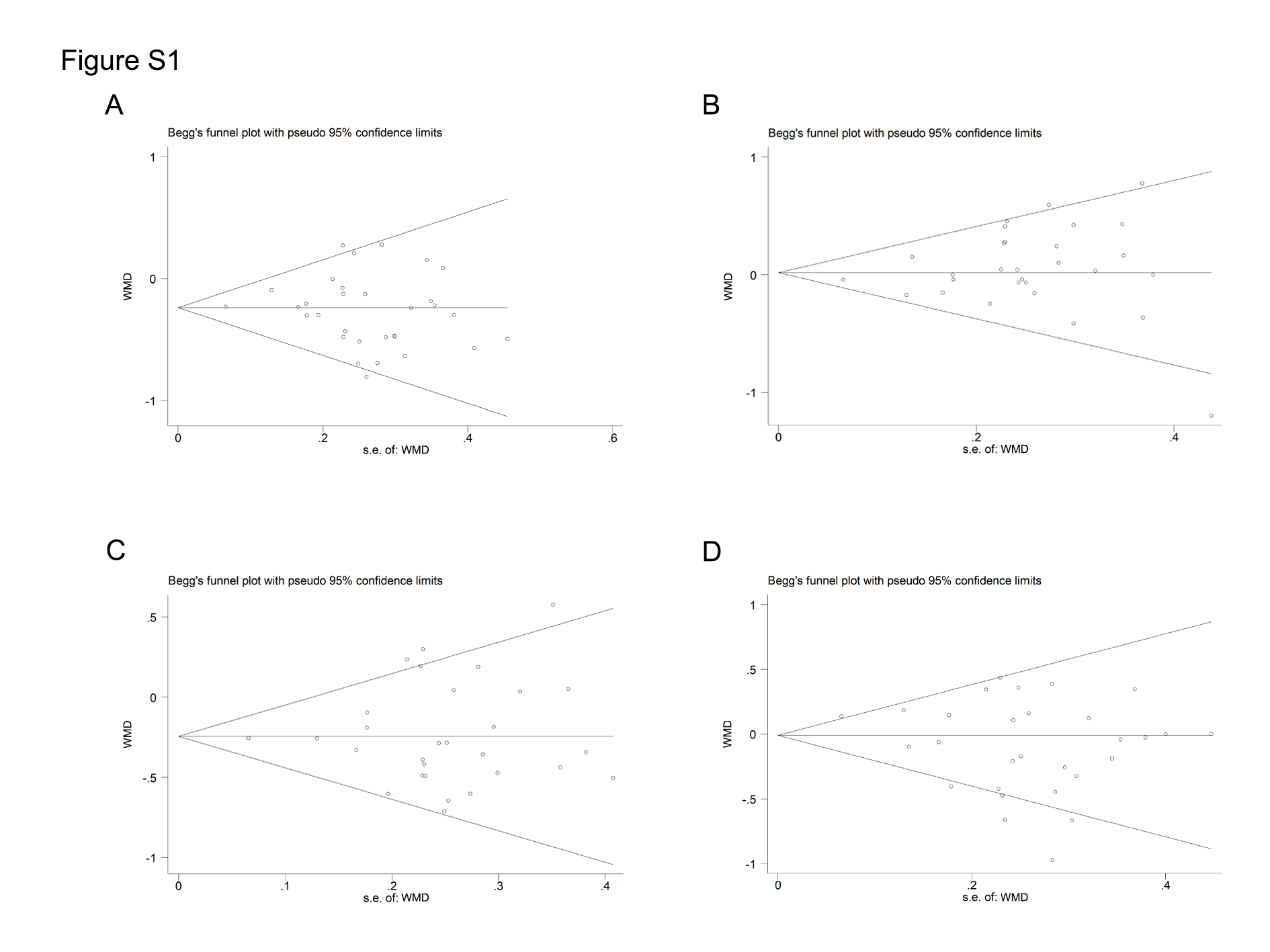

Supplement: Supplementary file 2 — Additional file 2: Figure S1. A. Funnel plot of green tea supplementation and total cholesterol. B. Funnel plot of green tea supplementation and LDL cholesterol. C. Funnel plot of green tea supplementation and HDL cholesterol. D. funnel plot of green tea supplementation and triglyceride. [file 12937_2020_557_MOESM2_ESM.tif]
